# L'endométriose de la paroi abdominale: à propos d'un cas rare

**DOI:** 10.11604/pamj.2013.15.86.2905

**Published:** 2013-07-04

**Authors:** Sofia Jayi, Meriem Laadioui, Hakima Bouguern, Hikmat Chaara, Aabdelilah Melhouf

**Affiliations:** 1 Service de gynécologie-obstétrique 2, CHU Hassan II de Fès, Université Sidi Mohammed Benabdellah, Maroc

**Keywords:** Endométriose, paroi abdominale, diagnostic, traitement, pronostic, prévention, endometriosis, abdominal wall, diagnosis, treatment, prognosis, prevention

## Abstract

L'endométriose de la paroi est une entité clinique rare, dont la physiopathologie demeure imprécise. Elle survient le plus souvent après une intervention chirurgicale gynécologique ou obstétricale. Nous rapportons le cas d'une patiente présentant une douleur cyclique, au niveau de la cicatrice de césarienne. Avec à l'examen clinique une masse de 3 cm localisée au niveau de la fosse iliaque droite. L'échographie-doppler a objectivé une image d'allure tissulaire, polylobée, à vascularisation centrale, mesurant 32/16 mm, évoquent une masse endométriosique ou une tumeur des parties molles. D'où la décision d'excision de la lésion, au cours de laquelle on a découvert une masse dure de 3/2 cm, en sous aponévrotique accolée au muscle grand droit de l'abdomen. L'étude anatomopathologique a confirmé le diagnostic d'endométriose pariétale. Les suites postopératoires étaient simples avec un recul de 2 ans et demie sans récidive de la masse ni de la douleur. A travers notre cas, nous insisterons sur les caractéristiques de cette pathologie notamment pronostic, ce qui permettra au praticien de comprendre l'intérêt du diagnostic et prise en charge précoce de cette entité - pour laquelle on ne pense jamais assez devant une masse pariétale - et de sa prévention au cours de chaque chirurgie gynécologique ou obstétricale.

## Introduction

La présence de la muqueuse endométriale en dehors de son site habituel (cavité utérine) définit l'endométriose externe. La localisation pariétale est une entité rare. Elle survient le plus souvent sur les cicatrices d'interventions chirurgicales avec hystérotomie, et touche 0,03 à 0,4 % des cicatrices de césarienne [1]. Elle fait partie de la faible proportion des endométrioses considérées comme iatrogènes [2] et dont l'étiopathogénie demeure imprécise [3]. Nous rapportons un nouveau cas d'endométriose localisée à la paroi abdominale, à travers lequel et à la lumière d'une revue de la littérature nous insisterons sur toutes les caractéristiques de cette entité notamment pronostic ce qui permettra au praticien de comprendre l'intérêt du diagnostic et prise en charge précoce de cette entité -pour laquelle on ne pense jamais assez devant une masse pariétale- et de sa prévention au cours de chaque chirurgie gynécologique.

## Patient et observation

**Figure 1:**
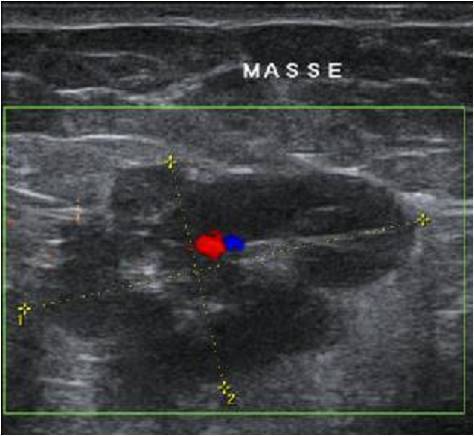
L'échographique montrant une image hypoéchogène hétérogène à contours irréguliers, et vascularisée au doppler couleur

Mme T.L âgée de 36 ans mariée et mère d'une fille, présentant une douleur exquise cyclique, cataméniale au niveau de la cicatrice de césarienne (type Pfannestiel), évoluant depuis une année et demie (soit 3ans et demie après la césarienne). L'examen physique a objectivé une masse de 3 cm localisée au niveau de la fosse iliaque droite au-dessus de la cicatrice de césarienne alors que le reste de l'examen était sans particularité. L'échographie des parties molles a objectivé la présence d'une image pariétale d'allure tissulaire polylobée, présentant une vascularisation centrale artério-veineuse au doppler couleur, mesurant 32/16 mm (Figure 1), faisant discuter Une masse endométriosique ou une tumeur des parties molles. D'où la décision d'excision de la lésion, au cours de laquelle on a découvert une masse dure de 3/2 cm, en sous aponévrotique accolée au muscle grand droit de l'abdomen. L'étude anatomopathologique a confirmé le diagnostic d'endométriose pariétale. Les suites postopératoires étaient simples avec une bonne évolution et un recul de 2ans et demie sans récidive de la masse ni de la douleur.

## Discussion

L'endométriose extra-génitale de la paroi abdominale est une entité rare. Elle ne représenterait que 0,03 à 2 % des endométrioses extra-génitales [2–4]. Dans la grande majorité des cas elles sont de localisation cicatricielle suite à une intervention chirurgicale, qu'elle soit cælioscopique sur les divers sites d'insertion des trocarts - ou par laparotomie [2–5], ou même après amniocentèse [2–6]. Toutes les interventions gynécologiques prédisposent à cette pathologie mais celles comportant une hystérectomie et surtout la morcélation utérine exposent à un risque majoré [2–6]. Cependant certains cas d'endométriose pariétale primitive ont été rapportés surtout au niveau de l'ombilic, des muscles droits et de la région inguinale secondairement à une dissémination par voie vasculaire ou lymphatique [2]. Alors que La localisation cicatricielle serait due à l'implantation directe des cellules endométriales suivie d'une inflammation secondaire induite probablement par des facteurs immunologiques et par un potentiel élevé de développement de ces cellules endométriales aux zones non épithélialisées [3–7].

L'endométriose pariétale se manifeste classiquement par une triade — comme c'est le cas de notre patiente: masse palpable, ferme de taille variable [2]; latéralisée par rapport à la ligne médiane et adhérente au fascia; avec des douleurs cycliques et une exacerbation cataméniales [2, 7, 8]. Le délai d'apparition reste très variable, allant de 2 mois à 15 ans après l'acte chirurgicale [2] et certaines se voient même après la ménopause par réactivation des foyers sous l'effet du traitement hormonal substitutif ou en présence d'une tumeur æstrogèno-secrétante [7]. Certains diagnostics différentiels peuvent être discutés cliniquement à savoir l'hématome, l'abcès, la hernie, l'éventration et les tumeurs quand la masse est à distance de la cicatrice; ainsi que le granulome cicatriciel et l'éventration si la masse est en regard de la cicatrice [7].

Une fois l'endométriose pariétale est suspectée, Le premier examen à demander reste l'échographie avec doppler couleur peut montrer une image qui peut être hypo-échogène hyper-vascularisée ‘comme ça a été retrouvé dans notre cas- [2], mixte ou liquidienne, dont les contours sont spiculés parfois hyper-échogénes selon la phase du cycle menstruel, la répartition entre les éléments du stroma et glandulaires et l'importance de la réaction inflammatoire. L'IRM constitue l'examen clé en montrant le signal particulier de l'hémorragie dans l'endométriome [2–9]; nodule iso ou hypo-intense en T1 et T2 ponctué de foyers hyper-intense en T1 et T2 [7].

La ponction aspiration ou la biopsie percutanée ont été rapportées par certains auteurs 2013 mais elles devraient être bannît vu le risque de dissémination le long du trajet de la ponction [7]-. La confirmation du diagnostic repose sur l'étude histologique du matériel excisé quand elle montre la présence de glandes endométriales, cependant dans certains cas le diagnostic différentiel se discute avec l'adénocarcinome ou les métastases d'adénocarcinome [3–7]. Cette exérèse constitue en même temps le gold standard du traitement et devrait passer bien au large de la lésion avec mise en place de plaque prothétique en cas de défet aponévrotique important [2, 7, 9]. Un traitement médical post-opératoire — par analogue de la LHRH ou DANAZOL — a été proposé, mais son bénéfice n'a pas encore été démontré [2]. La photocoagulation au laser CO2 a aussi été rapportée comme étant efficace mais reste peu répandue [7].

Les récidives ne sont pas rares, pouvant atteindre 10 à 15 %. Le taux de récidive est corrélé à la taille et à la profondeur de la lésion. Une étude, non randomisée, a cependant conclut qu'un traitement préopératoire, semble diminuer le taux de récidive [2] Par ailleurs certains cas de cancérisation d'endométriose de la paroi ont été rapportées dans la littérature ce qui justifie l'exérèse systématique de telles lésions [7–10]. Comparé au cancer de l'ovaire sur endométriose sous-jacente, le pronostic de cette complication d'une cicatrice abdominale est plutôt sombre. Dans la littérature, le taux de survie atteint 57 % avec un rec ul court de 20 mois et la forme histologique la plus courante est le carcinome à cellules claires, suivie du carcinome endométrioïde [10].

La prévention en cas de laparotomie est basée sur le lavage abondant de la cavité abdominale et de la cicatrice en fin d'intervention ainsi que le changement de gants pour le temps de fermeture pariétale, alors qu'en c'lioscopie, l'extraction des pièces opératoires dans un sac de protection et le lavage abondant de la cavité pelvienne devraient être systématiques. Ainsi, ces mesures relèvent de la bonne pratique chirurgicale bien que leur bénéfice n'a jamais été démontré [2].

## Conclusion

Nous attirons l'attention des praticiens sur le grand intérêt d'évoquer le diagnostic d'endométriose pariétale chaque fois qu'une patiente présente une masse de la paroi avec douleur cyclique, dans les suites proches ou lointaines d'une chirurgie gynécologique. ce qui devrait systématiquement conduire à une exérèse large en raison du risque de cancérisation dont la pronostic est considéré comme sombre.
